# Effect of Green Space Environment on Air Pollutants PM2.5, PM10, CO, O_3_, and Incidence and Mortality of SARS-CoV-2 in Highly Green and Less-Green Countries

**DOI:** 10.3390/ijerph182413151

**Published:** 2021-12-13

**Authors:** Sultan Ayoub Meo, Faris Jamal Almutairi, Abdulelah Adnan Abukhalaf, Adnan Mahmood Usmani

**Affiliations:** 1 Department of Physiology, College of Medicine, King Saud University, Riyadh 11461, Saudi Arabia; Faris11300@gmail.com (F.J.A.); Abdulelahabukhalaf@gmail.com (A.A.A.); 2 Diabetic Unit, College of Medicine, King Saud University, Riyadh 11461, Saudi Arabia; adnan_mahmood71@hotmail.com

**Keywords:** green space, non-green space, environmental pollution, SARS-CoV-2, COVID-19

## Abstract

Worldwide, over half of the global population is living in urban areas. The metropolitan areas are highly populated and environmentally non-green regions on the planet. In green space regions, plants, grass, and green vegetation prevent soil erosion, absorb air pollutants, provide fresh and clean air, and minimize the burden of diseases. Presently, the entire world is facing a turmoil situation due to the COVID-19 pandemic. This study investigates the effect of the green space environment on air pollutants particulate matter PM2.5, PM10, carbon monoxide (CO), ozone (O_3_), incidence and mortality of Severe Acute Respiratory Syndrome Coronavirus (SARS-CoV-2) in environmentally highly green and less-green countries. We randomly selected 17 countries based on the Environmental Performance Index (EPI) data. The 60% of the EPI score is based on seven categories: “biodiversity and habitat, ecosystem, fisheries, climate change, pollution emissions, agriculture, and water resources”. However, 40% of the score is based on four categories: “air quality, sanitation and drinking water, heavy metals, and waste management”. The air pollutants and SARS-CoV-2 cases and deaths were recorded from 25 January 2020, to 11 July 2021. The air pollutants “PM2.5, PM10, CO, and O_3_” were recorded from the metrological websites, Air Quality Index-AQI, 2021. The COVID-19 daily cases and deaths were obtained from the World Health Organization. The result reveals that air pollutants mean values for PM2.5 110.73 ± 1.09 vs. 31.35 ± 0.29; PM10 80.43 ± 1.11 vs. 17.78 ± 0.15; CO 7.92 ± 0.14 vs. 2.35 ± 0.03 were significantly decreased (*p* < 0.0001) in environmentally highly green space countries compared to less-green countries. Moreover, SARS-CoV-2 cases 15,713.61 ± 702.42 vs. 3445.59 ± 108.09; and deaths 297.56 ± 11.27 vs. 72.54 ± 2.61 were also significantly decreased in highly green countries compared to less-green countries. The green environment positively impacts human wellbeing. The policymakers must implement policies to keep the living areas, surroundings, towns, and cities clean and green to minimize air pollution and combat the present pandemic of COVID-19.

## 1. Introduction

Worldwide over half of the population, 4.2 billion (56%) inhabitants, live in urban areas. The rapid growth in population and urbanization resulted in an increasing proportion of the people living in metropolitan cities and caused an imbalance to provide healthy and sustainable living environments [1]. The swift urbanization and industrialization have limited public access to nature and increased exposure to air pollution and allied diseases. Worldwide, urban areas face challenging problems of growing populations, limited resources, and the increasing impact of rapid climate change. Urbanization and industrialization polluted the normal biological ecosystem, affected weather conditions, and caused an imbalance between the pattern of health and disease [2].

Worldwide, the total forest area is about 4.06 billion hectares, it covers 31% of the global land area, but forests are not equally distributed around the globe [3]. Deforestation, forest degradation, and wildfires continue to occur at alarming rates; it significantly decreases global biodiversity. During 2015–2020, the deforestation rate was 10 million hectares per year, considerably minimizing green spaces globally [3].

The green space environment, plants, parks, playgrounds, or vegetation in public and private places can provide natural opportunities to nature and biodiversity. Natural green spaces mitigate environmental hazards, air pollution, extreme weather events, heatwaves, excessive rainfall, or flooding [4]. The green space environment enhances ecological air quality, decreases air and noise pollution, and risks various diseases. It offers natural solutions to minimize environmental pollution, increase urban settings’ quality, promote healthy lifestyles, and improve the health and wellbeing of the residents [5].

The environmental green space is a component of “green infrastructure”. It is an integral part of public health-promoting settings. The green space areas with plants, trees, and gardens have been linked to less environmental pollution, healthy climate, and weather conditions. However, non-green space areas cause desert, sandstorms, ecological pollution, and adverse effects on human health [6]. Worldwide, mainly the urban population is facing a challenging issue of environmental pollution and allied health diseases. Environmental pollution has become a leading cause of transmission and pathogenesis of various bacterial and viral infections, including SARS-CoV-2 cases and deaths [6,7]. Since Dec 2019, worldwide, people have faced a challenging issue of “Severe Acute Respiratory Syndrome (SARS-CoV-2), also known as the COVID-19 pandemic”. It causes global physical, psychological, educational, and economic losses. As per the World Health Organization report, on 26 November 2021, there were 259, 502, 031 cases of COVID-19 and 5,183,003 (1.99%) deaths [8].

The scientific community established some linkage between air pollutants and SARS-CoV-2 cases and deaths [9], but no single study has been published on the impact of natural green space on the epidemiological trends of the COVID-19 pandemic. The present study hypothesized that a green space environment decreases the air pollutants and COVID-19 pandemic. Therefore, the present study investigates the SARS-CoV-2 cases and deaths among people living in ecologically highly green and less-green space countries.

## 2. Research Methodology

### 2.1. Selection of Highly Green and Less-Green Space Countries

The present study was conducted in the “Department of Physiology, College of Medicine, King Saud University, Riyadh, Saudi Arabia”. For this study, we randomly selected 17 countries based on the “Environmental Performance Index (EPI)” data of the countries of biological and natural ecosystem sustainability worldwide. Total 32 performance indicators were used across the 11 concerning categories. The EPI ranks the countries on environmental health, green regions, and ecosystem vitality [10].

### 2.2. EPI Scoring System of Environmentally Green Space and Less-Green Countries

The EPI classified the grading of the individual countries based on the various environmental factors. The environmental health policy and ecosystem vitality objective measure how healthy countries preserve, protect, and enhance natural ecosystems and green areas and protect people from environmental health risk factors. The 60% of the EPI score is based on seven categories: biodiversity (plant- and animal life on the earth) and habitat (natural environment), ecosystem, pollution productions, climate change, fisheries, agriculture, and water resources. However, 40% of the score was based on four categories: air quality, heavy metals, sanitation and drinking water, and waste management. As per “Environmental Performance Index-EPI” 2021 [10], we randomly selected ten environmentally highly green countries: Luxembourg, Finland, Norway, Denmark, Austria, Switzerland, Sweden, Germany, United Kingdom, and France. We also selected 07 environmentally less-green countries; these include Egypt, Thailand, Kuwait, Saudi Arabia, Indonesia, Brazil, and India [10].

### 2.3. Measurement of “PM2.5, PM10, CO, and O_3_, and SARS-CoV-2 Cases and Deaths”

After selecting the countries, the data on air pollutants “particulate matter PM2.5, PM10, carbon monoxide (CO), ozone (O_3_) pollutants, and SARS-CoV-2 cases and deaths” were documented from 25 January 2020 to 11 July 2021. The data was obtained from the first case of SARS-CoV-2 in these countries from 25 January to 11 July 2021. This study period of about one year and six months covers the various changes in the environment, weather conditions, and ups and downs in the epidemiological trends of the COVID-19 pandemic. The environmental pollutants “PM2.5, PM10, CO, and O_3_” were documented from the appearance of the first case reported in these states. The data on air pollutants, daily “PM2.5, PM10, CO, and O_3_” levels were recorded from the metrological websites, “Air Quality Index-AQI, 2021” [11], during the same period. However, the “data on COVID-19 daily cases and deaths were recorded from the official website of the World Health Organization (WHO)” for coronavirus [8]. For the confirmation of the data, the third co-investigator re-checked all the data.

### 2.4. Statistical Analysis and Ethical Statement

The statistical analysis was performed by using the SPSS software version 22.0, Chicago, USA, for Microsoft windows. The “mean values with standard deviation (SD) were calculated using a paired sample *t*-test”. The Pearson analysis was executed to predict the association of the air pollutant PM2.5, PM10, CO, and O_3_ on the number of SARS-CoV-2 daily cases and deaths. A *p*-value less than 0.05 was considered statistically significant. For this study, the “data on the daily new cases and deaths due to COVID-19 pandemic, particulate matter PM2.5, PM10, CO, and O_3_ allied information were obtained from the publicly available databases; hence ethical approval was not required”.

## 3. Results

**Figure 1:**
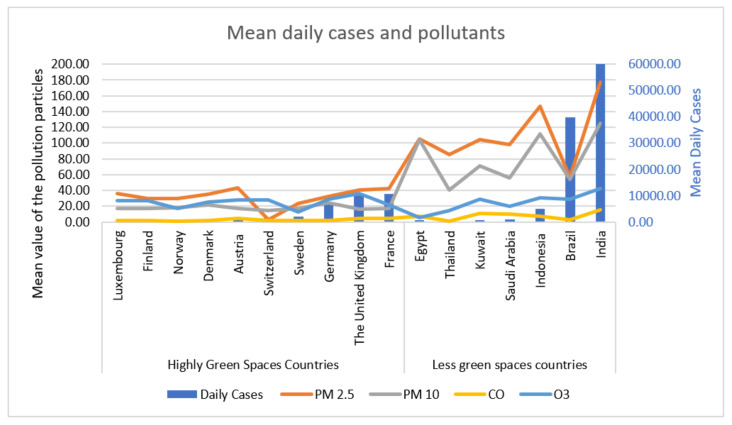
Environmental pollutants PM2.5, PM10, CO, O_3_, and daily cases due to SARS-CoV-2 in highly green and less-green space countries.

**Figure 2:**
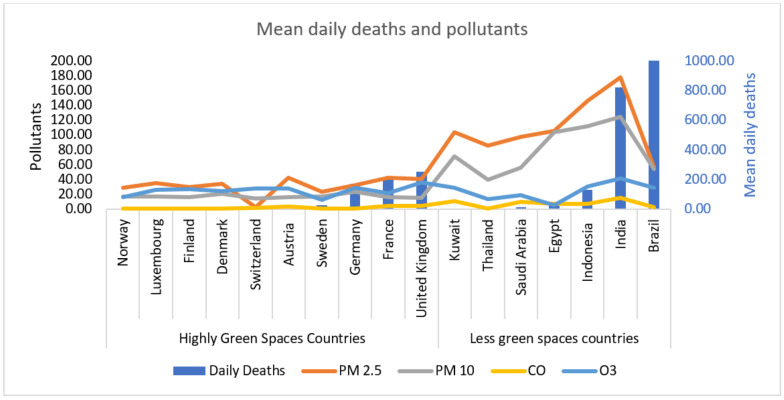
Environmental pollutants PM2.5, PM10, CO, O_3_, and daily deaths due to SARS-CoV-2 in highly green and less-green space countries.

**Figure 3:**
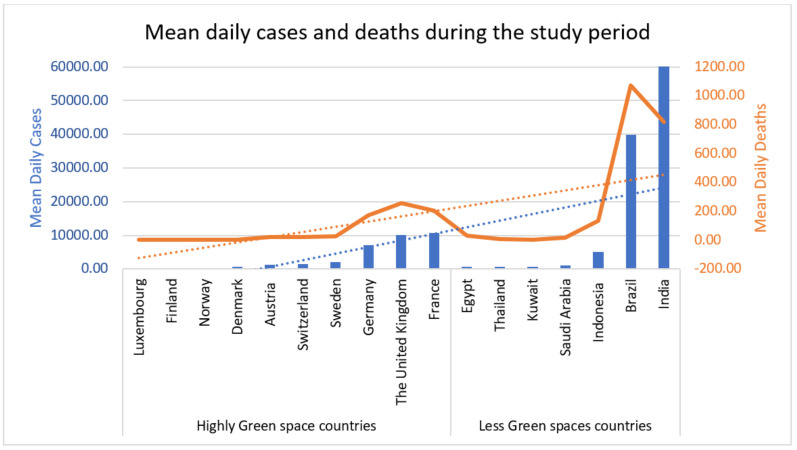
SARS-CoV-2 daily cases and deaths in highly green and less-green space countries.

Table 1 shows the environmentally greenspace countries with their EPI score, Luxembourg, Finland, Norway, Denmark, Austria, Switzerland, Sweden, Germany, United Kingdom, and France. Table 1 also shows the environmentally less-green space countries, including Egypt, Thailand, Kuwait, Saudi Arabia, Indonesia, Brazil, and India (Table 1). Table 1 also demonstrates the study period of all these countries, environmental pollutants, PM2.5, PM10, CO, O_3_, and SARS-CoV-2 daily cases and deaths in environmentally highly green and less-green space countries (Figure 1, Figure 2 and Figure 3).

The air pollutants particulate matter mean values for PM2.5 110.73 ± 1.09 vs. 31.35 ± 0.29; PM10 80.43 ± 1.11 vs. 17.78 ± 0.15; CO 7.92 ± 0.14 vs. 2.35 ± 0.03 were significantly decreased (*p* = 0.0001) in environmentally green countries compared to less-green countries (Table 1). Moreover, SARS-CoV-2 cases 15,713.61 ± 702.42 vs. 3445.59 ± 108.09; deaths 297.56 ± 11.27 vs. 72.54 ± 2.61 were also significantly decreased in green space countries compared to less-green countries (Table 2). However, no significant difference was noticed between O_3_ and SARS-CoV-2 daily cases and deaths in environmentally highly green and less-green space countries (Table 2). Environmental pollutants PM2.5, PM10, CO, O_3_, and SARS-CoV-2 daily cases and daily deaths are presented in (Figure 1 and Figure 2) in environmentally highly green and less-green space countries.

**Figure 4:**
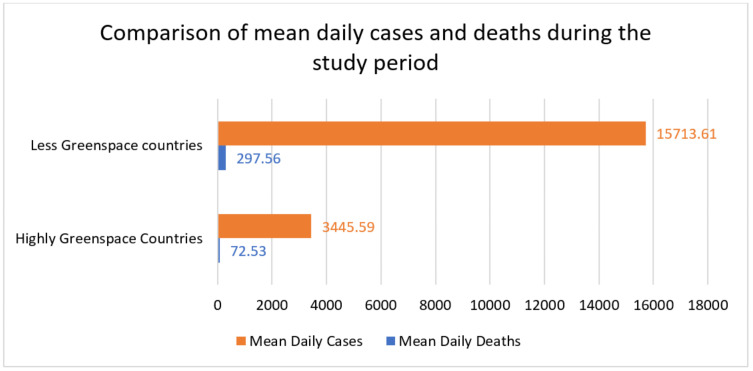
Comparison of mean daily cases and daily deaths during the study period.

**Figure 5:**
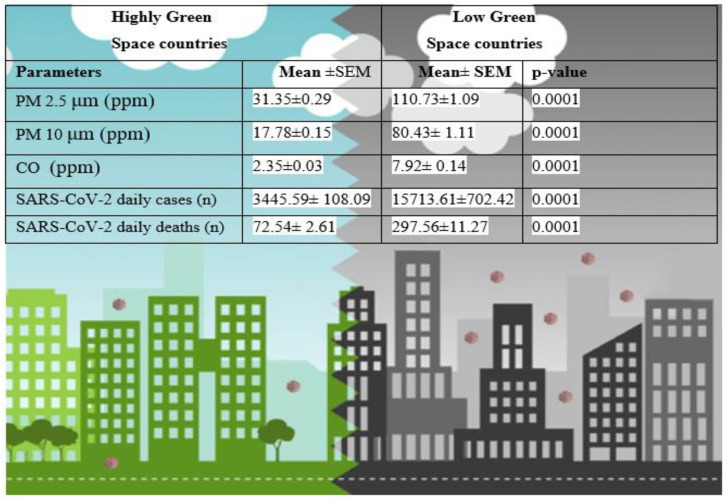
Graphical presentation and comparison of environmental pollutants PM2.5, PM10, CO, O_3_, and SARS-CoV-2 cases and deaths in highly green and less-green space countries.

Table 3 demonstrates the Pearson analysis outcomes. The definite dependent variable depends on air pollutants, and it was employed to envisage the pollutant parameters and the number of SARS-CoV-2 cases and deaths. The finding demonstrates that an increase in “PM2.5, PM10, CO, and O_3_” was allied to a significant rise in the number of SARAS-CoV-2 cases and deaths (*p* = 0.0001) (Table 3). Figure 4 compares mean daily cases and daily deaths from 25 January 2020, to 11 July 2021. Figure 5 reveals the graphical presentation and comparison of environmental pollutants and SARS-CoV-2 cases and deaths in highly green and less-green space countries.

Since the population is associated with the air pollutant parameters, it might be the reason for an increase in daily cases and daily deaths. Therefore, binary logistic regression analysis was applied to exclude the effect of population size confounder, considering that the average population of each country remained the same throughout the study period, and the adjusted odds ratio was calculated for daily cases and deaths. The analysis showed that after adjusting the confounding factor, the population size of each country, there is 1.0% (OR = 1.0) significantly (*p* < 0.001) increase in daily cases and 0.99% significantly (*p* < 0.001) increases in daily deaths because of high pollutants in non-green space countries (Table 4).

## 4. Discussion

Worldwide, a large percentage of the population dwells in urban areas, along with considerable benefits to health, education, and economies; however, urbanization has brought significant challenges for the social and natural biological systems. The quality of the urban ecosystem mainly depends on the residential setup, population, and availability of green spaces. The green areas are essential for natural biological productivity, environmental eminence and promote human health [12]. The green spaces provide ecological benefits by negating “urban heat, offsetting greenhouse gas emissions, and attenuating stormwater”. The green areas have direct health benefits by giving opportunities to the people for social interaction, physical activities, and psychological restoration [13]. In green space regions, plants, trees, grass, and other green vegetation provide wildlife habitat, prevent soil erosion, absorb and shield air pollutants, and provide fresh and clean air and minimize disease burden. The environmentally green space regions offer the community a happy and healthy living environment [14,15,16].

The swift worldwide urbanization with extreme weather-related conditions intensifies the effect of environmental threats such as air pollution, sandstorms, and heatwaves [17,18]. Environmental pollution has developed a threatening situation, particularly in the urban parts of the planet, where it generates a condition commonly called “background contamination” [19]. The epidemiologic studies have established an association among day-to-day differences in health outcomes, daily incidence and deaths, and variations in ambient particulate matter (PM) levels. Recent literature highlights that fine particulate air pollution has become an increasing problem for human health and risks hospital admission for cardiorespiratory diseases [20,21].

The green space value for human health has gained increasing attention during the present global pandemic of COVID-19. The pandemic has developed a highly hostile and frightening situation worldwide, and it caused considerable health and monetary losses globally [22]. The literature has established a link between PM pollution to the spread of COVID-19. Conticini et al., 2020 [23] performed a study in Italy and found that air pollution is considered an additional co-factor for causative high levels of lethality recorded more in the polluted area. In environmentally contaminated regions, about 12% of infected patients died compared to an average of 6.4% globally.

The scientific literature highlights that ecological pollutants enhance the spread of SARS-CoV-2 [24]. Zheng et al., 2001 [25] demonstrated that exposure to “NO_2_, PM2.5, and PM10” was related to a rise of about 38%, 32.0%, and 14.0% in SARS-CoV-2 cases, respectively. The results further highlight that atmospheric pollution has a linkage to SARS-COV-2 infection vulnerability to the population.

Zhu et al., 2020 [26] found a positive linkage between “PM2.5, PM10, CO, and O_3_” with SARS-CoV-2 disease in China. Coccia, 2021 [27] conducted a study in Italy, found that about 75% of individuals infected and 81% of deaths during the first wave of COVID-19 pandemic were in industrial areas with high air pollution. Furthermore, Qaid et al., 2021 [28] reported that PM2.5 has a relationship with the COVID-19 infected cases. Similarly, Bashir et al., 2020 [29] established a relationship and found that “PM10, PM2.5, SO_2_, NO_2_, and CO were linked with the COVID-19 epidemic in California”. Consistently, Chakrabarty et al., 2002 [30] identified that PM2.5 pollutants cause people more vulnerable to SARS-CoV-2 infection in the various states of the USA. Congruently, Paital and Agrawal, 2020 [31] also found that air impurities increase the risk of COVID-19 disease.

More recently, Meo and Abukhalaf et al., 2021 [7] steered a study on the impact of “PM2.5, CO, and O_3_ on the incidence and mortality of SARS-COV-2 infection in California, USA”. The authors identified that PM2.5 and CO were linked with increasing SARS-COV-2 cases and deaths in San Francisco. Another study piloted by Meo and colleagues et al., 2021 [9] reported that “PM2.5, CO, and O_3_” positively correlate with SARS-CoV-2 daily cases and deaths in London, UK. These studies support the supposition that “air pollutants PM2.5, PM10, and CO are connected with SARS-CoV-2 daily cases and deaths. The present study result reveals that air pollutants significantly decreased in environmentally green countries compared to less-green countries. Moreover, SARS-CoV-2 cases and deaths were significantly low in highly green countries compared to less-green countries. The findings support the hypothesis that plants, trees, and vegetation minimize the air pollutants and decrease the SARS-CoV-2 cases and deaths; results have an important message that nature positively impacts human wellbeing. It is vital to discuss the mechanisms behind the green space and how it can reduce the SARS-CoV-2 cases and deaths.

Roviello and colleagues [32] demonstrated that the pandemic’s severity was lower in southern Italy, with more forest per hectare. The lowest mortality rates were in Molise and Basilicata regions, where the forest was higher. The results further suggest that green forests and shrubland plants could protect the southern population. In another study, Roviello and colleagues [33] reported that the percentage of deaths per population was lower in greener areas such as Sardinia, Calabria, and Basilica versus northern regions with low forest coverage Lombardy and Emilia Romagna.

### Possible Mechanism of How SARS-COV-2 Cases and Deaths Are Low in Green Space Regions

The present study findings established some mechanisms for a thoughtful understanding of the impact of green space and its lowering impact on SARS-COV-2 cases and deaths. It is a fact that SARS-CoV-2 can spread with fine, ultrafine air pollutants on which the virus particles lie. The air pollutants get the surface particles and swiftly conveyances them long-distance [34]. The lungs’ exposure to air pollutants causes lung damage due to “oxidative stress, macrophage disfunction, and a disrupted epithelial barrier”. These factors drive increased lung damage [26,27] and increased SARS-C0V2 deaths. The pathophysiology of SARS-CoV-2 is mainly due to “redox-active components of air pollutants, oxidative mechanisms, and ACE2 overexpression underlying air pollution-exacerbated SARS-CoV-2 transmission” [35]. Moreover, air pollutants PM pollutants act as haulers of the virus, impair immunity and cause individuals to be susceptible to pathogens [20]. These pieces of evidence patronage the premise that air pollutants can play a role in spreading the SARS-CoV-2 infection [20,36]. The importance of this study is the use of plants, trees, green space as “scavengers” of particulate matter. In green areas, plants, vegetation performs essential ecological functions by removing various air pollutants [37].

McDonald et al., 2007 [38] reported that an increase in tree cover up to 54% would reduce air pollutants PM concentration by 26% by removing 200 tons of particulate matter per year. Similarly, increasing tree cover from 3.6% to 8% would reduce the pollutants by 2% from the entire environment. The health impact of urban green areas against particulate matter can be relevant because once the air particles flow in a turbulent, they hit a plant, trees, leaves, and adhere through dry deposition [39,40,41], and plant leaves can easily absorb the air pollutants.

According to study findings available in scientific literature, 1 m^2^ of leaf area can absorb 70 mg and 2.8 g of particulate matter per year [40,42]. It has also been reported that one hectare of trees, with 11% plant planting, removed 9.7 kg of pollution in a year [43] and removed the whole city area around 600 km^2^, 591 tons. Yang et al., 2005 [44] showed that plants in central Beijing removed about 1241 tons of particulate matter per annum. In another study, Nowak et al., 2013 [45] demonstrated the removal of PM2.5 from the trees in various 10 US cities. The quantity of PM2.5 removed annually from trees varies from 4.7 tons in NY to 64.5 tons in Atlanta. Ozdemir 2019 [46] determined the impact of roadside plants on vehicle-related PM2.5 and heavy metals. The authors identified that roadside PM2.5 levels were significantly reduced by 17%, and maximum removal of heavy metals. This function of absorbing the air pollutants by plants, trees and minimizing air pollution has significant value and community benefits by reducing human diseases and mortality. This is the fact that nature positively impacts human wellbeing.

## 5. Study Strengths and Limitations

This is the first novel study exploring the impact of the green space environment on air pollutants, “PM2.5, PM10, CO, O_3_, and SARS-CoV-2 daily cases and deaths”. It also helps to deepen the knowledge of how the plants, trees, and green vegetation minimize the air pollutants and the SARS-CoV-2 cases and deaths and prevent people from the COVID-19 pandemic. A limitation of this study is that SARS-CoV-2 cases and deaths may be altered for other factors such as temperature, humidity, population density, health care system, face mask, social distancing, and other air pollutants.

## 6. Conclusions

The air pollutants PM2.5, PM10, CO, and SARS-CoV-2 cases and deaths significantly decreased in environmentally highly green countries compared to less-green countries. The plants, grass, vegetation could capture the air pollutants and lower the air pollutants and SARS-CoV-2 cases and deaths. The study verdicts have an important message to policymakers and the public about the health impact of people living in environmentally green regions and its association with air pollutants and SARS-CoV-2 cases and deaths. The policymakers must implement strict policies to keep the living environment, surroundings, towns, and cities clean and green to minimize air pollution and combat against various diseases, including the COVID-19 pandemic.

## Figures and Tables

**Table 1:** Environmental pollutants, PM2.5, PM10, CO, O_3_, SARS-CoV-2 daily cases, and deaths in highly green and less-green space countries.

Countries	EPI Score	Data Collection Period	Cases Mean ± SD	Deaths Mean ± SD	PM2.5 Mean ± SD	PM10 Mean ± SD	CO Mean ± SD	O_3_ Mean ± SD
Highly Green Space Countries (*n* = 10)								
Denmark	82.5	27 February 2020 to 11 July 2021	596.04 ± 33.25	5.07 ± 0.37	32.99 ± 0.51	21.30 ± 0.51	1.41 ± 0.47	24.90 ± 0.37
Luxembourg	82.3	1 March 2020 to 11 July 2021	147.50 ± 9.09	1.64 ± 0.11	35.70 ± 0.66	17.21 ± 0.30	1.42 ± 0.04	26.58 ± 0.54
Switzerland	81.5	24 February 2020 to 11 July 2021	1449.46 ± 88.69	20.35 ± 1.38	2.99 ± 0.06	14.26 ± 0.45	1.89 ± 0.08	27.78 ± 0.55
United Kingdom	81.3	24 February 2020 to 11 July 2021	10,098.98 ± 624.04	254.76 ± 16.88	40.65 ± 1.31	15.97 ± 0.45	4.41 ± 0.15	25.18 ± 0.65
France	80.0	24 January2020 to 11 July 2021	10,642.99 ± 631.98	206.30 ± 12.12	42.26 ± 1.33	16.65 ± 0.45	4.41 ± 0.16	21.60 ± 0.56
Austria	79.6	26 February 2020 to 11 July 2021	1289.00 ± 77.50	20.79 ± 1.46	43.06 ± 1.09	16.84 ± 0.46	4.22 ± 0.16	27.97 ± 0.55
Finland	78.9	27 February 2020 to 11 July 2021	195.77 ± 9.99	1.94 ± 0.17	29.87 ± 0.59	16.61 ± 0.48	1.30 ± 0.04	27.22 ± 0.40
Sweden	78.7	31 January 2020 to 11 July 2021	2069.32 ± 115.48	27.58 ± 1.47	23.77 ± 0.66	16.99 ± 0.57	1.52 ± 0.04	23.95 ± 0.350
Norway	77.7	26 February 2020 to 11 July 2021	264.53 ± 18.55	1.59 ± 0.16	29.39 ± 0.58	17.75 ± 0.43	1.21 ± 0.06	16.79 ± 0.32
Germany	77.2	28 January 2020 to 11 July 2021	7036.09 ± 389.81	171.80 ± 11.85	32.35 ± 0.48	24.01 ± 0.63	1.59 ± 0.05	28.96 ± 0.49
Less-Green Space Countries (*n* = 07)								
Kuwait	53.6	3 February 2020 to 11 July 2021	752.62 ± 22.48	4.22 ± 0.16	104.38 ± 1.82	71.49 ± 2.45	10.92 ± 0.24	29.01 ± 0.66
Brazil	51.2	3 February 2020 to 11 July 2021	39,871.37 ± 1276.99	1069.79 ± 40.43	57.07 ± 1.38	54.37 ± 1.66	3.04 ± 0.11	28.75 ± 0.56
Thailand	45.4	3 February 2020 to 11 July 2021	676.71 ± 71.72	5.45 ± 0.64	85.68 ± 2.75	40.38 ± 0.80	1.15 ± 0.07	13.91 ± 0.38
Saudi Arabia	44.0	3 February 2020 to 11 July 2021	1006.20 ± 47.66	16.02 ± 0.60	97.80 ± 1.63	55.83 ± 2.55	9.97 ± 0.36	19.53 ± 0.80
Egypt	43.3	3 February 2020 to 11 July 2021	569.37 ± 20.43	32.96 ± 1.03	105.59 ± 1.75	104.22 ± 3.07	7.37 ± 0.22	5.85 ± 0.14
Indonesia	37.8	3 February 2020 to 11 July 2021	5072.28 ± 268.98	133.45 ± 6.16	146.85 ± 3.28	111.91 ± 3.59	7.51 ± 0.24	30.69 ± 1.08
India	27.6	3 February 2020 to 11 July 2021	62,046.71 ± 4133.54	821.00 ± 50.88	177.74 ± 3.78	124.78 ± 3.87	15.48 ± 0.74	42.08 ± 3.10

**Table 2:** Comparison between environmental pollutants PM2.5, PM10, CO, O_3_, and SARS-CoV-2 cases and deaths in highly green and less-green space countries.

Low Green Space Countries (*n* = 07)		Highly Green Space Countries (*n* = 10)	
Parameters	Mean ± SEM	Mean ± SEM	*p*-Value
PM 2.5 μm (ppm)	110.73 ± 1.09	31.35 ± 0.29	0.0001
PM 10 μm (ppm)	80.43 ± 1.11	17.78 ± 0.15	0.0001
CO (ppm)	7.92 ± 0.14	2.35 ± 0.03	0.0001
O_3_ (DU)	24.26 ± 0.51	25.09 ± 0.15	0.613
SARS-CoV-2 daily cases (*n*)	15,713.61 ± 702.42	3445.59 ± 108.09	0.0001
SARS-CoV-2 daily deaths (*n*)	297.56 ± 11.27	72.54 ± 2.61	0.0001

**Table 3:** Pearson correlation between environmental pollutants, PM2.5, PM10, CO, O_3_, and SARS-CoV-2 cases and deaths in highly green and less-green space countries.

Parameters	Low Green Space Countries (*n* = 7)		Highly Green SPACE Countries (*n* = 10)	
Pollutants	Daily Cases	Daily Deaths	Daily Cases	Daily Deaths
PM2.5 μm (ppm)	0.256 * (0.0001)	0.118 * (0.0001)	0.203 * (0.0001)	0.137 * (0.0001)
PM10 μm (ppm)	0.159 * (0.0001)	0.095 * (0.0001)	0.108 * (0.0001)	0.101 * (0.0001)
CO (ppm)	0.174 * (0.0001)	0.071 * (0.0001)	0.434 * (0.0001)	0.345 * (0.0001)
O_3_ (DU)	0.343 * (0.0001)	0.288 * (0.0001)	0.114 * (0.0001)	0.046 * (0.007)

* shows the level of significance.

**Table 4:** Binary logistic regression analysis for daily cases and daily deaths in low green space countries.

Variables	Adjusted Odds Ratios (OR)	95% CI	*p*-Value
Daily cases	1.0	1.00–1.00	*p* < 0.001 ***
Daily Deaths	0.99	0.998–0.99	*p* < 0.001 ***
Population	1.0	1.00–1.00	*p* < 0.001 ***

*** Significance = strongly significant at *p* < 0.05, CI = Confidence Interval.

## Data Availability

Data may be provided on reasonable request to the corresponding author.
